# Bis(2-butyl­imino­methyl-5-methoxy­phenolato-κ^2^ *N*,*O* ^1^)zinc(II)

**DOI:** 10.1107/S1600536809031109

**Published:** 2009-08-12

**Authors:** Jun Yang, Jin Li, Xian Zhang, Qiang Wang

**Affiliations:** a Engineering Research Center for Clean Production of Textile Dyeing and Printing, Ministry of Education, Wuhan 430073, People’s Republic of China

## Abstract

In the centrosymmetric title compound, [Zn(C_12_H_16_NO_2_)_2_], the Zn^II^ centre is coordinated by two *O*,*N*-bidentate Schiff base ligands, resulting in a slightly distorted *trans*-ZnN_2_O_2_ square-planar geometry for the metal ion. Two short intra­molecular C—H⋯O contacts occur in the mol­ecule.

## Related literature

For related structures, see: Zhu *et al.* (2003 ▶); You *et al.* (2003 ▶).
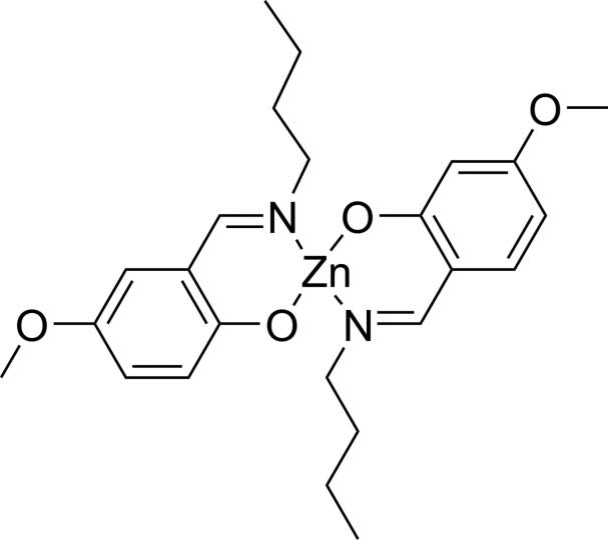


## Experimental

### 

#### Crystal data

[Zn(C_12_H_16_NO_2_)_2_]
*M* *_r_* = 477.89
Monoclinic, 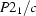

*a* = 13.250 (4) Å
*b* = 4.8845 (15) Å
*c* = 17.858 (5) Å
β = 93.555 (5)°
*V* = 1153.6 (6) Å^3^
*Z* = 2
Mo *K*α radiation
μ = 1.10 mm^−1^
*T* = 200 K
0.30 × 0.30 × 0.20 mm

#### Data collection

Bruker APEX CCD diffractometer
Absorption correction: multi-scan (*SADABS*; Bruker, 2000 ▶) *T*_min_ = 0.734, *T*_max_ = 0.811
13300 measured reflections
2856 independent reflections
2438 reflections with *I* > 2σ(*I*)
*R*_int_ = 0.155

#### Refinement

*R*[*F* ^2^ > 2σ(*F* ^2^)] = 0.058
*wR*(*F* ^2^) = 0.164
*S* = 1.10
2856 reflections
144 parameters
H-atom parameters constrained
Δρ_max_ = 1.13 e Å^−3^
Δρ_min_ = −0.67 e Å^−3^

### 

Data collection: *SMART* (Bruker, 2000 ▶); cell refinement: *SAINT* (Bruker, 2000 ▶); data reduction: *SAINT*; program(s) used to solve structure: *SHELXS97* (Sheldrick, 2008 ▶); program(s) used to refine structure: *SHELXL97* (Sheldrick, 2008 ▶); molecular graphics: *SHELXTL* (Sheldrick, 2008 ▶); software used to prepare material for publication: *SHELXTL*.

## Supplementary Material

Crystal structure: contains datablocks global, I. DOI: 10.1107/S1600536809031109/hb5031sup1.cif

Structure factors: contains datablocks I. DOI: 10.1107/S1600536809031109/hb5031Isup2.hkl

Additional supplementary materials:  crystallographic information; 3D view; checkCIF report

## Figures and Tables

**Table 1:** Selected bond lengths (Å)

Zn1—O1	1.8769 (17)
Zn1—N1^i^	2.004 (2)

Symmetry code: (i) 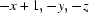.

**Table 2:** Hydrogen-bond geometry (Å, °)

*D*—H⋯*A*	*D*—H	H⋯*A*	*D*⋯*A*	*D*—H⋯*A*
C8—H8*B*⋯O1^i^	0.99	2.34	2.763 (3)	105
C9—H9*A*⋯O1^i^	0.99	2.55	3.087 (3)	114

Symmetry code: (i) 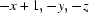.

**Fig. 1.**
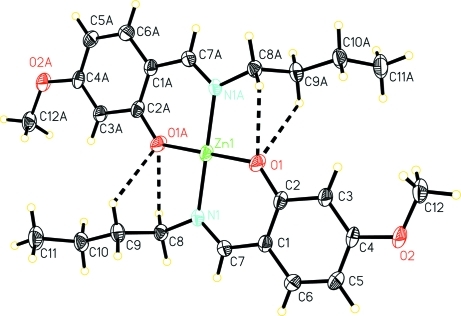
The molecular structure of (I). Displacement ellipsoids are drawn at the 30% probability level and dashed lines indicate hydrogen bonds. Atoms with a label A suffix are generated by the symmetry operator (1–x, 1–y, –z).

## supplementary crystallographic information

### Comment

As part of our ongoing studies of the structures of zinc(II) complexes (Zhu *et al.*, 2003; You *et al.*, 2003), we now report the crystal structure of the title compound, (I).

Compound (I) consists of a Zn^II^ atom and two bidentate salicylal schiff base ligands (Fig. 1). The central Zn^II^ atom exhibits 4-coordination by two N atoms from imine moieties and two O-anions from salicylal groups, forming a slightly distorted square planar geometry (Table 1).  Ring Zn1—N1—C7—C1—C2—O1 and Ring Zn1—N1A—C7A—C1A—C2A—O1A, togethor with the two benzene rings, stand in a plane with mean deviation of 0.0489 °.  Two short intramolecular C—H···O contacts occur in the molecule (Table 2).

### Experimental

0.5 mmol of zinc oxide, 1 mmol of 4-methoxysalicylaldehyde and 1 mmol of butylamine were dissolved in 10 ml methanol. After 3 ml ammonia was added, the result solution was then heated to 423 K for 10 h in a sealed vessel. The reactor was cooled to room temperature at a rate of 10 K h^-1^. The mixture was filtered and held at room temperature for 8 d. Colourless blocks of (I) were isolated in 47% yield.

### Refinement

All H atoms were placed in geometrically idealized positions (C—H = 0.95—0.99Å) and refined as riding with *U*_iso_(H) = 1.2U_eq_(C) or 1.5U_eq_(methyl C).

### Crystal data

**Table:**

[Zn(C_12_H_16_NO_2_)_2_]	*F*(000) = 504
*M**_r_* = 477.89	*D*_x_ = 1.376 Mg m^−^^3^
Monoclinic, *P*2_1_/*c*	Mo *K*α radiation, λ = 0.71073 Å
Hall symbol: -P 2ybc	Cell parameters from 2317 reflections
*a* = 13.250 (4) Å	θ = 2.7–26.5°
*b* = 4.8845 (15) Å	µ = 1.10 mm^−^^1^
*c* = 17.858 (5) Å	*T* = 200 K
β = 93.555 (5)°	Block, colorless
*V* = 1153.6 (6) Å^3^	0.30 × 0.30 × 0.20 mm
*Z* = 2	

### Data collection

**Table:**

Bruker APEX CCD diffractometer	2856 independent reflections
Radiation source: fine-focus sealed tube	2438 reflections with *I* > 2σ(*I*)
graphite	*R*_int_ = 0.155
φ and ω scans	θ_max_ = 28.4°, θ_min_ = 2.3°
Absorption correction: multi-scan (*SADABS*; Bruker, 2000)	*h* = −17→17
*T*_min_ = 0.734, *T*_max_ = 0.811	*k* = −6→6
13300 measured reflections	*l* = −23→23

### Refinement

**Table:**

Refinement on *F*^2^	Primary atom site location: structure-invariant direct methods
Least-squares matrix: full	Secondary atom site location: difference Fourier map
*R*[*F*^2^ > 2σ(*F*^2^)] = 0.058	Hydrogen site location: inferred from neighbouring sites
*wR*(*F*^2^) = 0.164	H-atom parameters constrained
*S* = 1.10	*w* = 1/[σ^2^(*F*_o_^2^) + (0.0665*P*)^2^ + 0.1817*P*] where *P* = (*F*_o_^2^ + 2*F*_c_^2^)/3
2856 reflections	(Δ/σ)_max_ < 0.001
144 parameters	Δρ_max_ = 1.13 e Å^−^^3^
0 restraints	Δρ_min_ = −0.67 e Å^−^^3^

### Special details

**Table:**

Geometry. All e.s.d.'s (except the e.s.d. in the dihedral angle between two l.s. planes) are estimated using the full covariance matrix. The cell e.s.d.'s are taken into account individually in the estimation of e.s.d.'s in distances, angles and torsion angles; correlations between e.s.d.'s in cell parameters are only used when they are defined by crystal symmetry. An approximate (isotropic) treatment of cell e.s.d.'s is used for estimating e.s.d.'s involving l.s. planes.
Refinement. Refinement of *F*^2^ against ALL reflections. The weighted *R*-factor *wR* and goodness of fit *S* are based on *F*^2^, conventional *R*-factors *R* are based on *F*, with *F* set to zero for negative *F*^2^. The threshold expression of *F*^2^ > σ(*F*^2^) is used only for calculating *R*-factors(gt) *etc*. and is not relevant to the choice of reflections for refinement. *R*-factors based on *F*^2^ are statistically about twice as large as those based on *F*, and *R*- factors based on ALL data will be even larger.

### Fractional atomic coordinates and isotropic or equivalent isotropic displacement parameters (Å^2^)

**Table:**

	*x*	*y*	*z*	*U*_iso_*/*U*_eq_	
C1	0.40796 (16)	0.2347 (5)	0.15609 (13)	0.0375 (5)	
C2	0.3582 (2)	0.0191 (5)	0.11694 (18)	0.0403 (6)	
C3	0.26490 (19)	−0.0732 (6)	0.14142 (16)	0.0437 (6)	
H3	0.2290	−0.2136	0.1143	0.052*	
C4	0.2254 (2)	0.0377 (5)	0.20399 (17)	0.0425 (6)	
C5	0.27511 (19)	0.2446 (6)	0.24413 (15)	0.0488 (6)	
H5	0.2475	0.3196	0.2875	0.059*	
C6	0.36504 (18)	0.3396 (6)	0.22021 (13)	0.0470 (6)	
H6	0.3995	0.4811	0.2479	0.056*	
C7	0.49911 (17)	0.3519 (5)	0.13234 (13)	0.0392 (5)	
H4	0.5281	0.4936	0.1631	0.047*	
C8	0.63958 (19)	0.4507 (5)	0.06201 (16)	0.0386 (5)	
H8A	0.6392	0.6192	0.0928	0.046*	
H8B	0.6393	0.5060	0.0087	0.046*	
C9	0.73453 (16)	0.2898 (5)	0.08264 (15)	0.0458 (6)	
H9A	0.7308	0.1105	0.0568	0.055*	
H9B	0.7391	0.2551	0.1374	0.055*	
C10	0.8289 (2)	0.4405 (6)	0.0612 (2)	0.0523 (7)	
H10A	0.8212	0.4914	0.0075	0.063*	
H10B	0.8362	0.6114	0.0909	0.063*	
C11	0.92355 (19)	0.2690 (8)	0.0748 (2)	0.0713 (10)	
H11A	0.9303	0.2131	0.1276	0.107*	
H11B	0.9828	0.3769	0.0628	0.107*	
H11C	0.9188	0.1059	0.0428	0.107*	
C12	0.0780 (2)	−0.2452 (6)	0.18909 (18)	0.0582 (8)	
H12A	0.0683	−0.1895	0.1364	0.087*	
H12B	0.0119	−0.2684	0.2101	0.087*	
H12C	0.1151	−0.4187	0.1924	0.087*	
N1	0.54697 (13)	0.2884 (4)	0.07401 (11)	0.0366 (4)	
O1	0.39291 (14)	−0.0997 (5)	0.05842 (11)	0.0542 (5)	
O2	0.13414 (17)	−0.0399 (4)	0.23031 (14)	0.0539 (6)	
Zn1	0.5000	0.0000	0.0000	0.0382 (2)	

### Atomic displacement parameters (Å^2^)

**Table:**

	*U*^11^	*U*^22^	*U*^33^	*U*^12^	*U*^13^	*U*^23^
C1	0.0311 (11)	0.0452 (13)	0.0370 (11)	0.0012 (10)	0.0067 (9)	0.0032 (9)
C2	0.0334 (14)	0.0431 (15)	0.0463 (16)	0.0002 (9)	0.0159 (11)	0.0018 (9)
C3	0.0340 (12)	0.0495 (13)	0.0491 (15)	−0.0069 (12)	0.0153 (10)	−0.0039 (12)
C4	0.0299 (13)	0.0518 (15)	0.0474 (16)	0.0030 (10)	0.0165 (11)	0.0061 (11)
C5	0.0444 (13)	0.0631 (17)	0.0407 (13)	0.0016 (12)	0.0176 (10)	−0.0046 (11)
C6	0.0440 (13)	0.0555 (16)	0.0424 (13)	−0.0034 (12)	0.0087 (10)	−0.0067 (11)
C7	0.0335 (11)	0.0431 (13)	0.0409 (12)	−0.0017 (10)	0.0023 (9)	−0.0012 (10)
C8	0.0331 (12)	0.0362 (11)	0.0472 (14)	−0.0047 (10)	0.0089 (10)	0.0018 (10)
C9	0.0311 (11)	0.0490 (14)	0.0578 (15)	−0.0058 (10)	0.0073 (10)	0.0079 (11)
C10	0.0332 (14)	0.0565 (15)	0.0681 (19)	−0.0094 (13)	0.0101 (12)	0.0000 (15)
C11	0.0345 (14)	0.086 (2)	0.094 (2)	−0.0009 (14)	0.0141 (15)	−0.0003 (19)
C12	0.0362 (13)	0.0653 (19)	0.075 (2)	−0.0047 (13)	0.0203 (13)	0.0043 (15)
N1	0.0275 (9)	0.0381 (10)	0.0446 (10)	−0.0016 (8)	0.0068 (7)	0.0031 (8)
O1	0.0458 (10)	0.0578 (12)	0.0625 (12)	−0.0190 (10)	0.0321 (9)	−0.0203 (10)
O2	0.0384 (11)	0.0685 (13)	0.0578 (13)	−0.0067 (9)	0.0265 (9)	−0.0022 (9)
Zn1	0.0311 (3)	0.0394 (3)	0.0457 (3)	−0.00210 (13)	0.0137 (2)	−0.00106 (14)

### Geometric parameters (Å, °)

**Table:**

C1—C2	1.405 (4)	C9—C10	1.521 (4)
C1—C6	1.406 (3)	C9—H9A	0.9900
C1—C7	1.425 (3)	C9—H9B	0.9900
C2—O1	1.304 (3)	C10—C11	1.515 (4)
C2—C3	1.411 (4)	C10—H10A	0.9900
C3—C4	1.374 (4)	C10—H10B	0.9900
C3—H3	0.9500	C11—H11A	0.9800
C4—O2	1.377 (3)	C11—H11B	0.9800
C4—C5	1.382 (4)	C11—H11C	0.9800
C5—C6	1.371 (3)	C12—O2	1.426 (4)
C5—H5	0.9500	C12—H12A	0.9800
C6—H6	0.9500	C12—H12B	0.9800
C7—N1	1.291 (3)	C12—H12C	0.9800
C7—H4	0.9500	N1—Zn1	2.004 (2)
C8—N1	1.488 (3)	Zn1—O1	1.8769 (17)
C8—C9	1.510 (3)	Zn1—N1^i^	2.004 (2)
C8—H8A	0.9900	Zn1—O1^i^	1.8769 (17)
C8—H8B	0.9900	Zn1—O1^i^	1.8769 (17)
			
C2—C1—C6	118.5 (2)	C10—C9—H9B	109.2
C2—C1—C7	122.2 (2)	H9A—C9—H9B	107.9
C6—C1—C7	119.3 (2)	C11—C10—C9	112.2 (3)
O1—C2—C1	123.7 (3)	C11—C10—H10A	109.2
O1—C2—C3	117.7 (2)	C9—C10—H10A	109.2
C1—C2—C3	118.6 (3)	C11—C10—H10B	109.2
C4—C3—C2	120.8 (3)	C9—C10—H10B	109.2
C4—C3—H3	119.6	H10A—C10—H10B	107.9
C2—C3—H3	119.6	C10—C11—H11A	109.5
C3—C4—O2	123.8 (3)	C10—C11—H11B	109.5
C3—C4—C5	121.1 (3)	H11A—C11—H11B	109.5
O2—C4—C5	115.1 (3)	C10—C11—H11C	109.5
C6—C5—C4	118.8 (2)	H11A—C11—H11C	109.5
C6—C5—H5	120.6	H11B—C11—H11C	109.5
C4—C5—H5	120.6	O2—C12—H12A	109.5
C5—C6—C1	122.2 (2)	O2—C12—H12B	109.5
C5—C6—H6	118.9	H12A—C12—H12B	109.5
C1—C6—H6	118.9	O2—C12—H12C	109.5
N1—C7—C1	127.7 (2)	H12A—C12—H12C	109.5
N1—C7—H4	116.1	H12B—C12—H12C	109.5
C1—C7—H4	116.1	C7—N1—C8	116.0 (2)
N1—C8—C9	111.7 (2)	C7—N1—Zn1	123.59 (15)
N1—C8—H8A	109.3	C8—N1—Zn1	120.33 (16)
C9—C8—H8A	109.3	C2—O1—Zn1	130.26 (18)
N1—C8—H8B	109.3	C4—O2—C12	117.3 (2)
C9—C8—H8B	109.3	O1^i^—Zn1—O1	180.0
H8A—C8—H8B	107.9	O1^i^—Zn1—N1^i^	91.74 (8)
C8—C9—C10	111.9 (2)	O1—Zn1—N1^i^	88.26 (8)
C8—C9—H9A	109.2	O1^i^—Zn1—N1	88.26 (8)
C10—C9—H9A	109.2	O1—Zn1—N1	91.74 (8)
C8—C9—H9B	109.2	N1^i^—Zn1—N1	180.0
			
C6—C1—C2—O1	−177.6 (3)	C9—C8—N1—C7	104.5 (3)
C7—C1—C2—O1	3.3 (4)	C9—C8—N1—Zn1	−77.6 (2)
C6—C1—C2—C3	3.2 (4)	C1—C2—O1—Zn1	−10.8 (4)
C7—C1—C2—C3	−175.9 (2)	C3—C2—O1—Zn1	168.5 (2)
O1—C2—C3—C4	178.1 (3)	C3—C4—O2—C12	−0.7 (4)
C1—C2—C3—C4	−2.6 (4)	C5—C4—O2—C12	177.6 (3)
C2—C3—C4—O2	179.0 (3)	C2—O1—Zn1—O1^i^	−103.5 (2)
C2—C3—C4—C5	0.8 (4)	C2—O1—Zn1—O1^i^	−103.5 (2)
C3—C4—C5—C6	0.3 (4)	C2—O1—Zn1—N1^i^	−169.8 (3)
O2—C4—C5—C6	−178.0 (2)	C2—O1—Zn1—N1	10.2 (3)
C4—C5—C6—C1	0.3 (4)	C7—N1—Zn1—O1^i^	175.0 (2)
C2—C1—C6—C5	−2.1 (4)	C8—N1—Zn1—O1^i^	−2.69 (17)
C7—C1—C6—C5	177.0 (2)	C7—N1—Zn1—O1^i^	175.0 (2)
C2—C1—C7—N1	1.5 (4)	C8—N1—Zn1—O1^i^	−2.69 (17)
C6—C1—C7—N1	−177.5 (2)	C7—N1—Zn1—O1	−5.0 (2)
N1—C8—C9—C10	172.7 (2)	C8—N1—Zn1—O1	177.31 (17)
C8—C9—C10—C11	−174.5 (3)	C7—N1—Zn1—N1^i^	104.5 (4)
C1—C7—N1—C8	178.5 (2)	C8—N1—Zn1—N1^i^	−73.1 (2)
C1—C7—N1—Zn1	0.8 (3)		

Symmetry codes: (i) −*x*+1, −*y*, −*z*.

### Hydrogen-bond geometry (Å, °)

**Table:**

*D*—H···*A*	*D*—H	H···*A*	*D*···*A*	*D*—H···*A*
C8—H8B···O1^i^	0.99	2.34	2.763 (3)	105
C9—H9A···O1^i^	0.99	2.55	3.087 (3)	114

Symmetry codes: (i) −*x*+1, −*y*, −*z*.

## References

[bb1] Bruker (2000). *SMART*, *SAINT* and *SADABS* Bruker Analytical X-ray Instruments Inc., Madison, Wisconsin, USA.

[bb2] Sheldrick, G. M. (2008). *Acta Cryst.* A**64**, 112–122. 10.1107/S0108767307043930 18156677

[bb3] You, Z.-L., Lin, Y.-S., Liu, W.-S., Tan, M.-Y. & Zhu, H.-L. (2003). *Acta Cryst.* E**59**, m1025–m1027.

[bb4] Zhu, H.-L., Meng, F.-J., Wang, Z.-D. & Yang, F. (2003). *Z. Kristallogr. New Cryst. S*truct. **218**, 321–322.

